# English Workshops with Simulated Patients and Peers Reduce Medical Students’ Apprehension About Speaking in English

**DOI:** 10.1007/s40670-024-02217-3

**Published:** 2024-11-06

**Authors:** Philippe Codron, Erwan Autret, Martine Fisbach, Kayleigh O’Sullivan, Helene Riveau, Cédric Annweiler, Nicolas Lerolle, Loïc Biere

**Affiliations:** 1 Health Faculty, University of Angers, 49045 Angers, France; 2 Department of Neurology, University Hospital of Angers, Angers, France; 3 Department of Geriatric Medicine and Memory Clinic, University Hospital of Angers, Angers, France; 4 Department of Anesthesia and Critical Care, University Hospital of Angers, Angers, France; 5 Department of Cardiology, University Hospital of Angers, Angers, France

**Keywords:** Medical English, Workshops, Simulation, Patients, Peers

## Abstract

In this study, we evaluated workshops with simulated patients and peers to improve English-speaking confidence among medical students. Results showed reduced apprehension when interviewing patients, high course satisfaction, perceived progress in English proficiency, and increased confidence in future interactions with patients, supporting simulation-based methods in medical English education.

**Supplementary Information:**

The online version contains supplementary material available at 10.1007/s40670-024-02217-3.

In the context of globalized health care, medical English skills are becoming increasingly important for physicians. Effective communication in English has become crucial not only for communicating effectively with non-French-speaking patients and international colleagues, but also for engaging with the vast amount of English-language medical literature, attending global conferences, and contributing to scientific journals. Traditional medical English training in France, compulsory since 1992 [1], focuses primarily on reading and writing, neglecting the practical linguistic and medical skills required for real-life interactions [2]. This gap prompted us to develop a novel, holistic approach centered on simulation-based workshops designed to integrate language skills with medical knowledge and practical application. Our goal was to reduce students’ anxiety when interacting with English-speaking patients and peers.

The workshops were added to the medical English courses at the Faculty of Medicine in Angers in 2020 for 4th year students. First, students were introduced to the concept via their online platform and asked to consult a glossary in preparation for the course. Simulation workshops were then organized at the Faculty of Medicine, in groups of ten students supervised by an English teacher and a physician. The sessions were conducted in English only, from start to finish. The sessions consisted of (1) interviewing standardized patients, (2) reporting the case and discussing it with peers, (3) communicating findings to the patient, and (4) receiving comprehensive feedback. Each session lasted 2 h and students participated twice a year. The program involved 3 English teachers and 12 English-speaking physicians.

To evaluate the effectiveness of this new teaching method, two questionnaires based on the Medical English Language Anxiety Scale (MELAS) [3] were used to measure students' apprehension in reading, writing, listening, and speaking in English (pre- and post-test, available in Supplementary Information, main parts reported in Table 1). The pre-course survey was administered during the first month of the semester (September 2022) before the workshops began, and the post-test was administered in the last month of the semester (May 2023). Since reading and writing were not part of the simulation, they were not included in the post-test. Of the 175 fourth-year medical students, 128 completed the two questionnaires, resulting in a response rate of 73.1%. Pre-test results revealed varying degrees of apprehension in reading, writing, listening, and speaking English (Table 1). Post-test results showed significant reductions in apprehension, particularly in listening a document in English (*p* < 0.001) and speaking with patients (*p* < 0.001), though public speaking anxiety remained unchanged (*p* = 0.31). At the end of the year, 92.7% of the students were satisfied with the course, and 73.5% felt they had made progress in their English. Finally, 76.6% of the students felt more confident about conducting an interview in English with a non-French-speaking patient in the future.

**Table 1:** Grouped results and pre–post comparisons

	Pre-test results	Post-test results	*p* (pre-/post-test)
Estimated personal English level (mean)	5.27/10	5.89/10	*p* < 0.001 *
*Reading*			
Have had the opportunity to read a medical or scientific text in English % (*n*)	100% (128/128)	-	-
Moderate or high apprehension about reading a document in English % (*n*)	18.0% (23/128)	-	-
*Writing*			
Have had the opportunity to write a medical or scientific text in English % (*n*)	14.1% (18/128)	-	-
Moderate or high apprehension about writing a document in English % (*n*)	32.8% (42/128)	-	-
*Listening*			
Have had the opportunity to listen a medical or scientific audio or video in English % (*n*)	65.6% (84/128)	-	-
Moderate or high apprehension about listening a document in English % (*n*)	30.4% (39/128)	18.7% (24/128)	*p* < 0.001 **
*Speaking*			
Have had spoken in English during an oral presentation in public % (*n*)	17.1% (22/128)	-	-
Moderate or high apprehension about speaking in English during an oral presentation % (*n*)	57.0% (73/128)	52.3% (67/128)	*p* = 0.31 **
Have had spoken with an English-speaking patient during their studies % (*n*)	35.1% (45/128)	-	-
Moderate or high apprehension about speaking with an English-speaking patient % (*n*)	31.2% (40/128)	27.3% (35/128)	*p* < 0.001 **

^*^Student’s *t*-test for paired, quantitative, normally distributed data. **Mc Nemar’s chi-squared test with continuity correction for paired proportions

Addressing English language apprehension in both general and medical contexts is crucial in the education of future medical professionals. Previous studies conducted in China to assess English communication among medical students show high anxiety levels (70–80%) [4, 5]. Our results indicate lower levels of apprehension, possibly due to the smaller linguistic gap between French and English, alongside differing educational curricula at the primary and secondary levels. Nevertheless, a significant proportion (about one-third) of students still experience considerable anxiety, especially during group discussions or patient interactions in English.

To mitigate this, workshops with simulated patients and peers train students through immersion in practical scenarios. This approach aims to integrate language skills with medical knowledge, diagnostic reasoning, decision-making, and interpersonal skills. The small-group format of these workshops encourages open discussion and active participation and is based on a near-peer teaching approach that avoids segregation by proficiency level. Both English language educators and medical professionals collaboratively design and rehearse scenarios, ensuring a comprehensive and dynamic learning environment.

In our experience, the course’s reception was predominantly positive and the results of our survey point in this direction, although it is important to note that there may be a bias associated with the self-reported nature of the data. Most students reported satisfaction, improved English proficiency, and increased confidence in conducting patient interviews in English. Despite its successes, the program faces organizational limitations, particularly in resource allocation. Indeed, the requirement of extensive teaching staff limits student participation to biannual workshops, insufficient for optimal results. This limitation was echoed in student feedback. Exploring alternative formats or additional resources could allow for more regular sessions. Additionally, the program excludes the development of reading and writing skills in English. Incorporating elements that also develop English literacy skills would allow for a more comprehensive approach to medical English education. Finally, the remaining apprehension in public speaking, which is probably related to broader factors, could also be addressed in other teaching modules through public speaking workshops, in French or English.

In conclusion, this study demonstrates the potential of a simulation-based approach to complement traditional medical English education, significantly reducing student’' apprehension in oral English communication and improving their confidence in future medical interactions. A randomized two-arm study contrasting traditional training with a combination of conventional and simulation-based training with skill assessment by English-language Objective Structured Clinical Examinations (OSCE) could provide a more comprehensive evaluation of this innovative teaching approach.

## Supplementary Information

Below is the link to the electronic supplementary material.

Supplementary file1 (DOCX 33 KB)

## Data Availability

The data that support the findings of this study are available from the corresponding author upon reasonable request.
