# Supplementary material for: English Workshops with Simulated Patients and Peers Reduce Medical Students’ Apprehension About Speaking in English
Source: Med Sci Educ. 2024 Nov 6;35(1):21–3. doi: 10.1007/s40670-024-02217-3 (PMC11933570; doi:10.1007/s40670-024-02217-3)
Supplement: Supplementary file 1 — Supplementary file1 (DOCX 33 KB) [file 40670_2024_2217_MOESM1_ESM.docx]

**Supplementary information**

**Questionnaire Anglais Médical 1 (Pre-test)**

Ce formulaire à remplir avant la première séance a pour but d'évaluer le sentiment de confiance ou d'appréhension que vous pouvez éprouver à l'idée de mener un interrogatoire médical ou restituer une situation clinique en Anglais. Le formulaire comprend 4 sections (lecture, écoute, écriture et conversation en Anglais). Les réponses à ce questionnaire seront anonymisées, nous comptons sur votre entière participation.

**Nom et prénom (les réponses seront anonymisées)**

**Comment évaluez-vous votre niveau d'Anglais médical ?**

Terrible 1 - 10 Excellent

**Etes-vous titulaire d'un diplôme d'Anglais (TOEFL, TOEIC, GMAT) ?**

Oui

Non

**1. Lecture de l'Anglais médical**

**Avez-vous déjà eu l'occasion de lire un texte en Anglais traitant de médecine ou de sciences au cours de votre cursus ? (article de journal, site internet, article scientifique, case-report, etc.)**

Oui

Non

**La perspective d'avoir à lire un texte en Anglais traitant de médecine ou de sciences est-elle source d'appréhension ?**

Oui un peu

Oui beaucoup

Oui énormément

Non pas du tout

2. Ecoute de l'Anglais médical

**Avez-vous déjà eu l'occasion d'écouter une présentation, une piste audio ou une vidéo traitant d'informations médicales ou scientifiques en Anglais au cours de votre cursus ? (Présentation en congrès, vidéo internet, enregistrement audio, film, etc.)**

Oui

Non

**La perspective d'avoir à écouter une présentation, une piste audio ou une vidéo traitant d'informations médicales ou scientifiques en Anglais est-elle source d'appréhension ?**

Oui un peu

Oui beaucoup

Oui énormément

Non pas du tout

3. Rédaction de l'Anglais médical

**Avez-vous déjà eu l'occasion d'écrire un texte médical ou scientifique en Anglais au cours de votre cursus ? (Article, case-report, abstract, poster, etc.)**

Oui

Non

**La perspective d'avoir à écrire un texte médical ou scientifique en Anglais est-elle source d'appréhension ?**

Oui un peu

Oui beaucoup

Oui énormément

Non pas du tout

4. Conversation en Anglais médical

**Avez-vous déjà eu l'occasion de conduire un entretien en Anglais avec un patient non francophone au cours de votre cursus ?**

Oui

Non

**La perspective d'avoir à converser en Anglais avec un patient Anglophone est-elle source d'appréhension ?**

Oui un peu

Oui beaucoup

Oui énormément

Non pas du tout

5. Prise de parole en public en Anglais

**Avez-vous déjà eu l'occasion de prendre la parole en Anglais lors d'une présentation orale en public au cours de votre cursus ? (Présentation d'un cas en staff médical en Anglais, d'un travail de recherche en congrès international, d'une situation clinique en visio, etc.)**

Oui

Non

**La perspective d'avoir à prendre la parole en Anglais lors d'une présentation orale en public est-elle source d'appréhension ?**

Oui un peu

Oui beaucoup

Oui énormément

Non pas du tout

**Questionnaire Anglais Médical 2 (Post-test)**

Ce court formulaire de 8 questions à remplir APRES LA DERNIERE SEANCE a pour but d'évaluer le sentiment de confiance que vous éprouvez désormais à l'idée de mener un interrogatoire médical ou restituer une situation clinique en Anglais. Les réponses à ce questionnaire seront anonymisées, nous comptons sur votre entière participation.

**Nom et prénom (les réponses seront anonymisées)**

**Comment évaluez-vous aujourd'hui votre niveau d'Anglais médical ?**

Terrible 1 – 10 Excellent

**La perspective d'avoir à écouter une présentation, une piste audio ou une vidéo traitant d'informations médicales ou scientifiques en Anglais est-elle source d'appréhension ?**

Oui un peu

Oui beaucoup

Oui énormément

Non pas du tout

**La perspective d'avoir à converser en Anglais avec un(e) patient(e) est-elle source d'appréhension ?**

Oui un peu

Oui beaucoup

Oui énormément

Non pas du tout

**La perspective d'avoir à prendre la parole en Anglais lors d'une présentation orale médicale est-elle source d'appréhension ?**

Oui un peu

Oui beaucoup

Oui énormément

Non pas du tout

**Êtes-vous satisfait(e) du format d’enseignement proposé** **par simulation d'une consultation en anglais ?**

Très insatisfait(e) 1 – 10 Très satisfait(e)

**Avez-vous le sentiment d'avoir progressé en Anglais (vocabulaire, fluence, compréhension) ?**

Aucune progression 1 – 10 Progression significative

**Vous sentez-vous plus confiant(e) pour mener un interrogatoire en Anglais à l'avenir ?**

Pas de différence 1- 10 Beaucoup plus confiant(e)

**Avez-vous d'éventuelle(s) remarque(s) ou proposition(s) pour améliorer et faire évoluer cet enseignement ?**
